# Retro-protein XXA is a remarkable solubilizing fusion tag for inclusion bodies

**DOI:** 10.1186/s12934-022-01776-7

**Published:** 2022-04-02

**Authors:** Xi Xie, Pei Wu, Xiaochen Huang, WenFeng Bai, Bowen Li, Ning Shi

**Affiliations:** 1 State Key Laboratory of Structural Chemistry, Fujian Institute of Research on the Structure of Matter, Chinese Academy of Sciences, 155 Yangqiao Road West, Fuzhou, 350002 China; 2 University of Chinese Academy of Sciences, Beijing, 100049 China; 3 Institute of Vascular Anomalies, Shanghai TCM-Integrated Hospital, Shanghai University of Traditional Chinese Medicine, 230 Baoding Road, Hongkou, Shanghai, 200082 China

**Keywords:** Inclusion bodies, Solubilizing fusion tag, Antifreeze protein (AFP), AXX, Retro-protein, XXA

## Abstract

**Background:**

Producing large amounts of soluble proteins from bacteria remains a challenge, despite the help of current various solubilizing fusion tags. Thus, developing novel tags is necessary. Antifreeze protein (AFP) has excellent solubility and hydrophilicity, but there are no current reports on its use as a solubilizing fusion tag. Additionally, there is no precedent for using retro-proteins (reverse sequence) as solubilizing fusion tags. Therefore, we selected the antifreeze protein AXX and obtained its retro-protein XXA by synthesizing the XXA gene for the development of a new solubilizing fusion tag.

**Results:**

XXA exhibits better stability and ease of expression than AXX; hence, we focused the development of the solubilizing fusion tag on XXA. XXA fused with the tested inclusion bodies, significantly increasing the soluble expression compared with commonly used solubilizing fusion tags such as GST, Trx, Sumo, MBP, and NusA. The tested proteins became soluble after fusion with the XXA tag, and they could be purified. They maintained a soluble form after XXA tag removal. Finally, we used enzymatic digestion reaction and western blot experiments to verify that bdNEDP1 and NbALFA, which were soluble expressed by fusion with XXA, were active.

**Conclusion:**

We developed the novel solubilizing fusion tag XXA, which could more effectively facilitate the soluble expression of inclusion bodies compared with current commonly used tags. XXA could function at both low and high temperatures, and its moderate molecular weight has a limited impact on the output. These properties make XXA an ideal fusion tag for future research and industrial production. Moreover, for the first time, we highlighted the broad potential of antifreeze protein as a solubilizing fusion tag, bringing retro-protein into practical application.

**Supplementary Information:**

The online version contains supplementary material available at 10.1186/s12934-022-01776-7.

## Background

At present, *Escherichia coli* remains the preferred expression system due to its advantages including large yield, low cost, mature technology, short cycle, and easy expansion for production [1]. However, the *E. coli* expression system has various shortcomings. One major problem is that target proteins often form incorrect aggregations or insoluble inclusion bodies [2]. Inclusion bodies are complex and their mechanisms of formation remain debated. Recently, it was discovered that inclusion bodies are rich in β-sheets and can be combined with amyloid-tropic dyes, suggesting that they are related to the formation of amyloids [3, 4].

To date, researchers have used fusion tags on protein expression to facilitate soluble expression of recombinant proteins, and a variety of tags have been developed including Spy [5], Flag [6], GST [7], Trx [8], Sumo [9], NusA [10], and MBP [11]. Although numerous commercial tags exist, many proteins are still too insoluble for use. Therefore, more efficient solubilizing fusion tags are necessary, which was the goal of our research.

Antifreeze proteins are generally rich in Thr and Ser, which can form hydrogen bonds with water molecules for provide ice binding sites [12] to exert their thermal hysteresis activity and other functions [13, 14]. Even antifreeze proteins (AFPs) that are hydrophobic, as a whole, are able to use twisted main-chain to form hydrogen bonds to bind multiple water molecules [15]. Hence, AFPs are an excellent candidate as a solubilizing fusion tag. However, there is no attempt has been reported that use AFPs as solubilizing fusion tag until now.

Native-proteins and the corresponding retro-proteins, inverso-proteins, and retroinverso-proteins are four unique isomers with mimetic sequences but different configurations [16]. Researchers have long considered whether these mimetics can fold into similar structures and whether they have functions. As a result, numerous studies have been conducted in this area, such as exploring retroinverso-proteins with anti-HIV representation [17] and inverso-protein, which can be assembled into mirror 5S ribosomes [18]. Although the peculiarities of retro-proteins with a reversed sequence have long been known [19, 20], after it was verified that retro-proteins can fold into a stable structure [21], research on retro-proteins has stalled and has not focused on practical application until recently.

Our research aims to develop a solubilizing fusion tag with strong solubility, wide applicability, and moderate molecular weight. Based on this goal, we chose an AFP from *Chlorella sorokiniana* as a fusion tag and named it AXX (Accession Number: PRW45461). We synthesized the retro-protein of AXX and found that it does not exist in nature but is an artificial protein, and we named this retro-protein XXA.

We initially tested whether the antifreeze protein AXX and the artificial protein XXA have the potential to act as solubilizing fusion tags. The results indicate that both tags have the function of promoting soluble expression. Next, we determined that the stability and expression of retro-protein XXA are better than that of antifreeze protein AXX. Combining these two factors, we used XXA for the development of a solubilizing fusion tag during follow-up. First, we demonstrated that XXA not only has a stronger effect than common solubilizing fusion tags, but also improves a variety of inclusion bodies. Second, we demonstrated that most inclusion bodies that were tested could be purified and remain soluble after removal of the XXA tag. Moreover, the protease bdNEDP1 and nanobody NbALFA, which were fused with XXA, were tested for activity. Finally, we proposed a hypothesis of the mechanism of XXA as a solubilizing fusion tag.

## Results

### The comparison of characteristics and solubilizing effects of XXA and AXX

Since knowledge of XXA and AXX is presently minimal, we were curious about the differences, if any, between AXX and XXA in terms of their properties and solubilizing effects. First, TMHMM v.2.0 and SignalP 5.0 Server were used to confirm that neither AXX nor XXA contains transmembrane and signal peptides. Partial properties of these were obtained through ProtParam software and numerous physicochemical properties of AXX and XXA were the same, with particularly high hydrophilicity (Additional file 1).

**Fig. 1:**
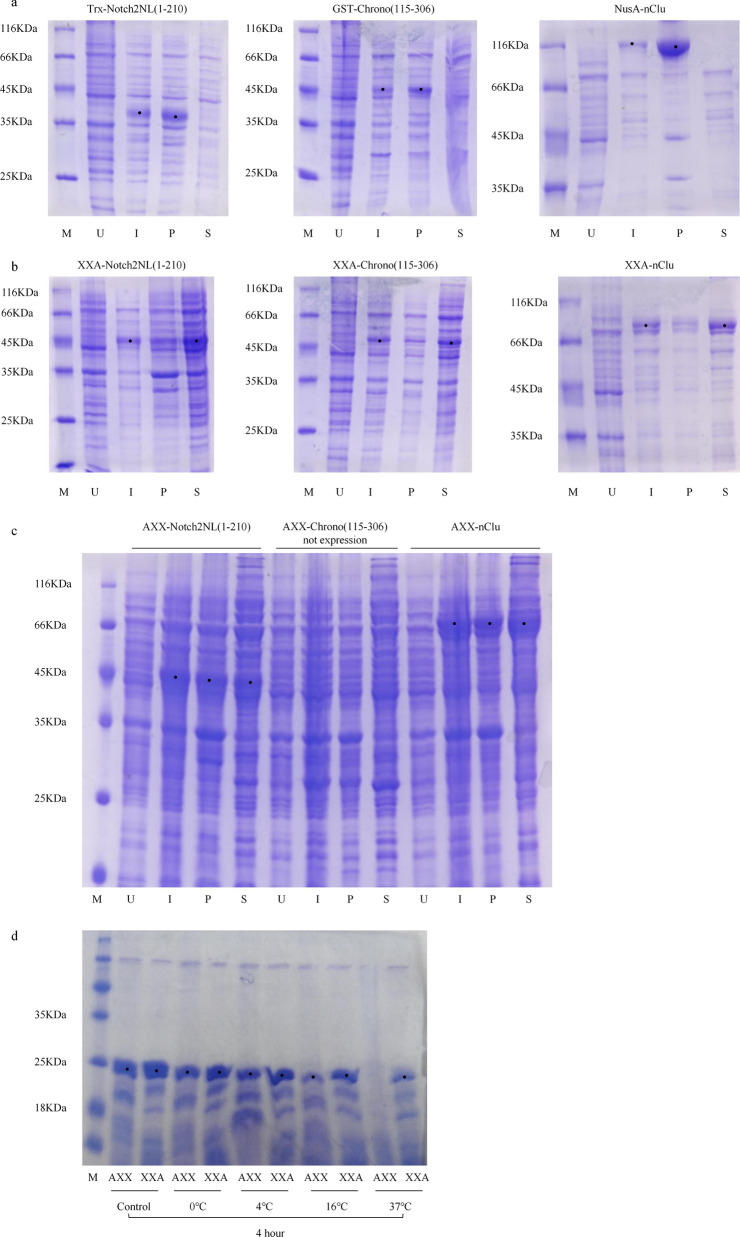
XXA and AXX have a strong solubilizing effect on different inclusion bodies, but XXA is more stable and easier to express. **a** The fusion proteins were inclusion bodies when Trx-Notch2NL(1–210), GST-Chrono(115–306), and NusA-nClu expression were at 16 °C. **b** XXA as a fusion tag clearly enhanced the soluble expression of Notch2NL(1–210), Chrono(115–306), and nClu at 16 °C. **c** AXX could improve the soluble expression of Notch2NL(1–210) and nClu, but AXX-Chrono(115–306) fusion protein could not express the same vector and situation. **d** The degradation of XXA was slower than that of AXX. *M* marker, *U* uninduced whole cells, *I* induced whole cells, *S* supernatant of the induced cells, *P* precipitate of the induced cells

Previous experiments in our laboratory found that three proteins, Chrono [22] (115–306), Notch2NL [23] (1–210), and nCLu [24, 25], were difficult to express in a soluble form using *E. coli*. The products were mainly located in the precipitate even when Chrono (115–306) was fused with a GST tag, when Notch2NL (1–210) was fused with a Trx tag, and when nClu was fused with a Nus tag (Fig. 1a). Therefore, we expressed these three proteins after fusion with XXA or AXX to observe whether XXA and AXX could promote the soluble expression of these three proteins. Sodium dodecyl sulphate–polyacrylamide gel electrophoresis (SDS-PAGE) results showed that Chrono (115–306), Notch2NL (1–210), and NusA were soluble when expressed and exist in the supernatant after being fused with XXA. These data indicate that XXA could significantly promote the soluble expression of these three inclusion body proteins, with XXA demonstrating a stronger solubilizing effect than GST, Trx, and NusA (Fig. 1b). The results of AXX showed that it can promote the soluble expression of Notch2NL (1–210) and nClu too. However, the AXX-Chrono (115–306) fusion protein was not expressed, but the XXA-Chrono (115–306) fusion protein was successfully expressed, despite the sequence of the vector being identical with the exception of the tag (Fig. 1c).

**Fig. 2:**
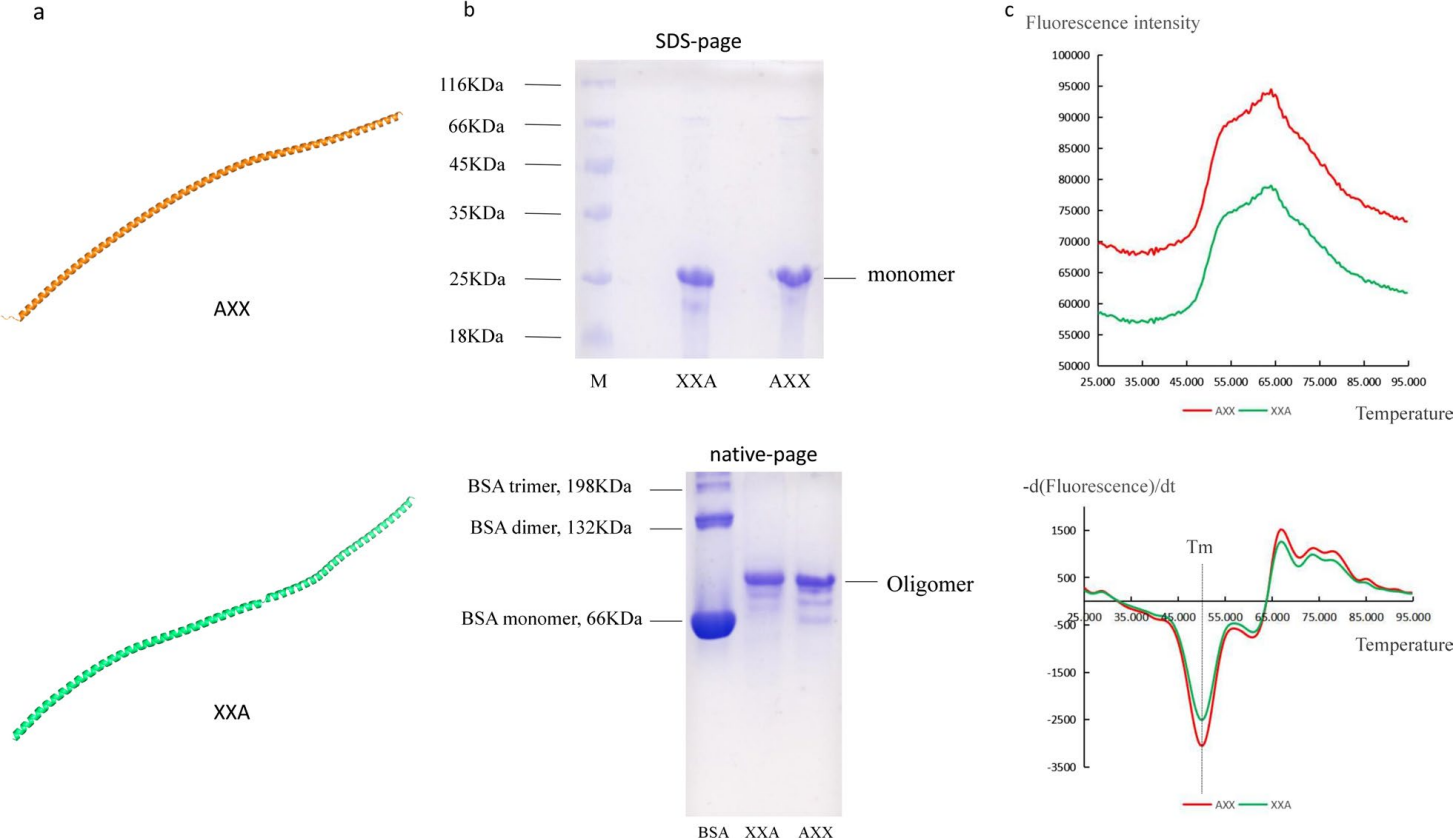
Structures and thermal stabilities of AXX and XXA.** a** The tertiary structures of AXX and XXA were predicted by tfold software. Both AXX and XXA are composed of long-segment α helices. **b** SDS-PAGE and Native-PAGE were used to study the aggregation states of AXX and XXA. **c** The results of the protein thermal shift experiment showcased that both AXX and XXA have high thermal stability and almost identical Tm values, suggesting that they may have similar structures

Next, we wanted to discover additional similarities and differences between XXA and AXX, since AXX-Chrono (115–306) was not expressed but XXA-Chrono (115–306) was successfully expressed and existed in a soluble state. Therefore, their secondary structures were predicted by NPS@_GORIV server (Additional file 2) and their tertiary structures were obtained use tFold server, which is based on the Free Modeling method (Fig. 2a). The results show that both proteins are mainly composed of long α-helices. Later, the quaternary structure information of XXA and AXX in aqueous solution were confirmed by Native-PAGE. The two protein bands in SDS-PAGE were approximately located at 25 kDa, while the two bands in Native-PAGE were at the same position and located between the bovine serum albumin (BSA) monomer and dimer (Fig. 2b). This suggests that XXA and AXX have the same aggregation state in solution, while both are limited oligomers rather than monomers or disordered high polymers.

Thermal stability is a key characteristic of protein for industrial use. Therefore, we studied the thermal stability of XXA and AXX. Thermal shift assays showed that both proteins not only have high thermal stability, but also have nearly identical T_m_ values (Fig. 2c). These results demonstrate that their high thermal stability makes them suitable for scientific experiments and industrial production, and their similar T_m_ values and aggregation state suggest that they may have very similar three-dimensional structures. However, the degradation rate experiments at different temperatures showed that the degradation of XXA was slower than that of AXX (Fig. 1d).

Thus, our follow-up experiments in the development of the solubilizing fusion tag focused on retro-protein XXA because it has better stability and expression than AXX.

### XXA may be a solubilizing fusion tag at low and high temperatures

We then expanded the kinds of inclusion bodies involved in this experiment and compared the enhancement effect of soluble expression with the commonly used sumo and MBP tags. Thus, we used NbALFA [26] and CUA63106 for expression experiments.

**Fig. 3:**
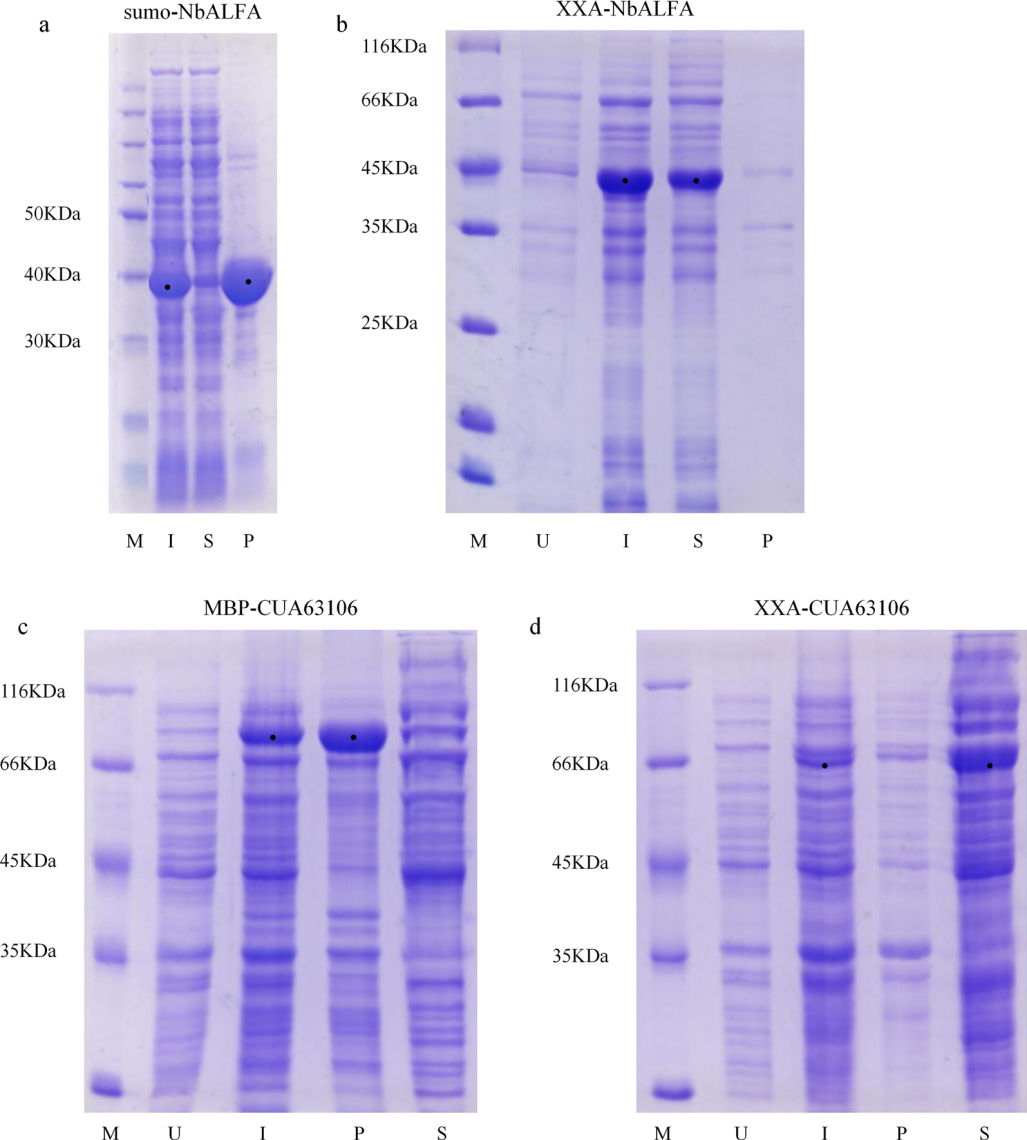
XXA exhibited its solubilizing effect at both high and low temperatures.** a** Most sumo-NbALFA fusion proteins were expressed in an insoluble form at 16 °C. **b** The NbALFA was expressed as soluble at 16 °C after fusion with XXA. **c** Most MBP-CUA63106 fusion proteins were expressed as insoluble at 25 °C. **d** The CUA63106 was expressed as soluble at 25 °C after fusion with XXA. *M* marker, *U* uninduced whole cells, *I* induced whole cells, *S* supernatant of the induced cells, *P* precipitate of the induced cells

Among these tags, the fusion protein of NbALFA and sumo was expressed at low temperature at 16 °C and the product was mainly located in the precipitate (Fig. 3a). Yet, when NbALFA and XXA were fused and expressed under the same conditions, most of the products existed in a soluble form (Fig. 3b). These results showed that XXA significantly promoted the soluble expression of NbALFA, and the effect was better than a sumo tag.

We next performed tested fusion proteins MBP-CUA63106 and XXA-CUA63106 at 25 °C (Fig. 3c). The results showed that the MBP-CUA63106 expressed at 25 °C was mainly in inclusion bodies, while the fusion protein XXA-CUA63106 expressed at a high temperature of 25 °C mostly existed in the supernatant (Fig. 3d). This indicates that XXA promotes the soluble expression of protein CUA63106, has good performance at a higher temperature of 25 °C, and is more effective than MBP in promoting the soluble expression of inclusion bodies.

### XXA, located at the N-terminus or C-terminus, may promote the soluble expression of heterologous proteins

We demonstrated that XXA, as a solubilizing fusion tag, has a good effect on promoting soluble expression in some inclusion bodies. However, in previous experiments, XXA was located at the N-terminus of other fusion proteins mentioned in this study. First, we wanted to further explore whether XXA could also promote soluble expression when it is located at the C-terminus. In addition, we wanted to further investigate whether XXA promotes the soluble expression on more types of inclusion bodies. Thus, we chose another protein, bdNEDP1 [27], for these experiments.

**Fig. 4:**
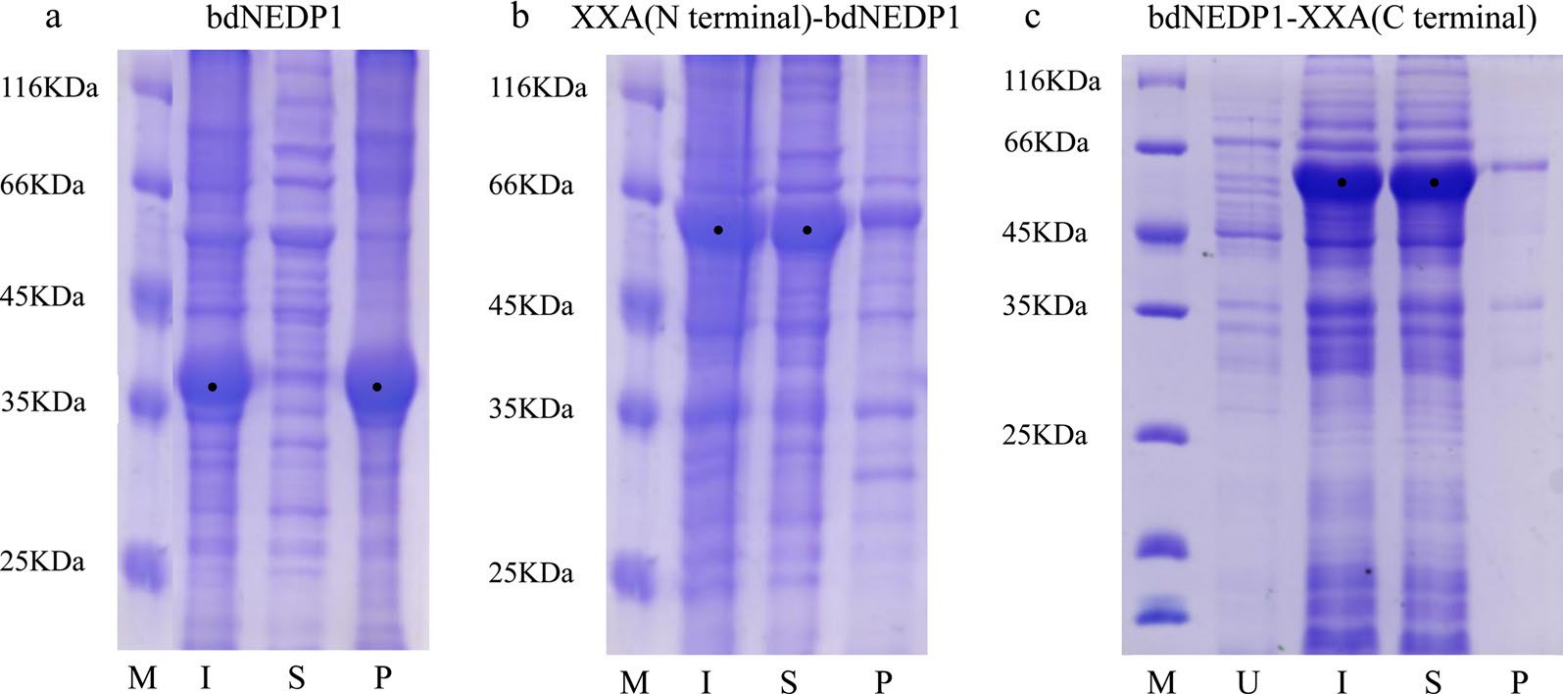
Either locate at N-terminal or C-terminal, XXA could promote the soluble expression of heterologous protein.** a** bdNEDP1 is an inclusion body when expressed alone at 16 °C. **b** bdNEDP1 was expressed in soluble form at 16 °C and was primarily located in solution after fusion with an N terminal XXA. **c** bdNEDP1 was expressed as soluble at 16 °C and primarily located in solution after fusion with a C terminal XXA. *M* marker, *U* uninduced whole cells, *I* induced whole cells, *S* supernatant of the induced cells, *P* precipitate of the induced cells

If bdNEDP1 was expressed alone, most of the proteins were located in the pellet (Fig. 4a). When XXA was located at the N-terminus and fused with bdNEDP1, most of the recombinant proteins were expressed in a soluble form (Fig. 4b). The products remained distributed in the supernatant, even if XXA was placed at the C-terminus (Fig. 4c). These findings indicate that XXA, as a fusion tag, can not only promote the soluble expression of bdNEDP1, but can exhibit its effect regardless of whether located at the N-terminus or C-terminus.

### Multiple comparisons of the effect of XXA and other tags on the soluble expression of heterologous proteins

**Fig. 5:**
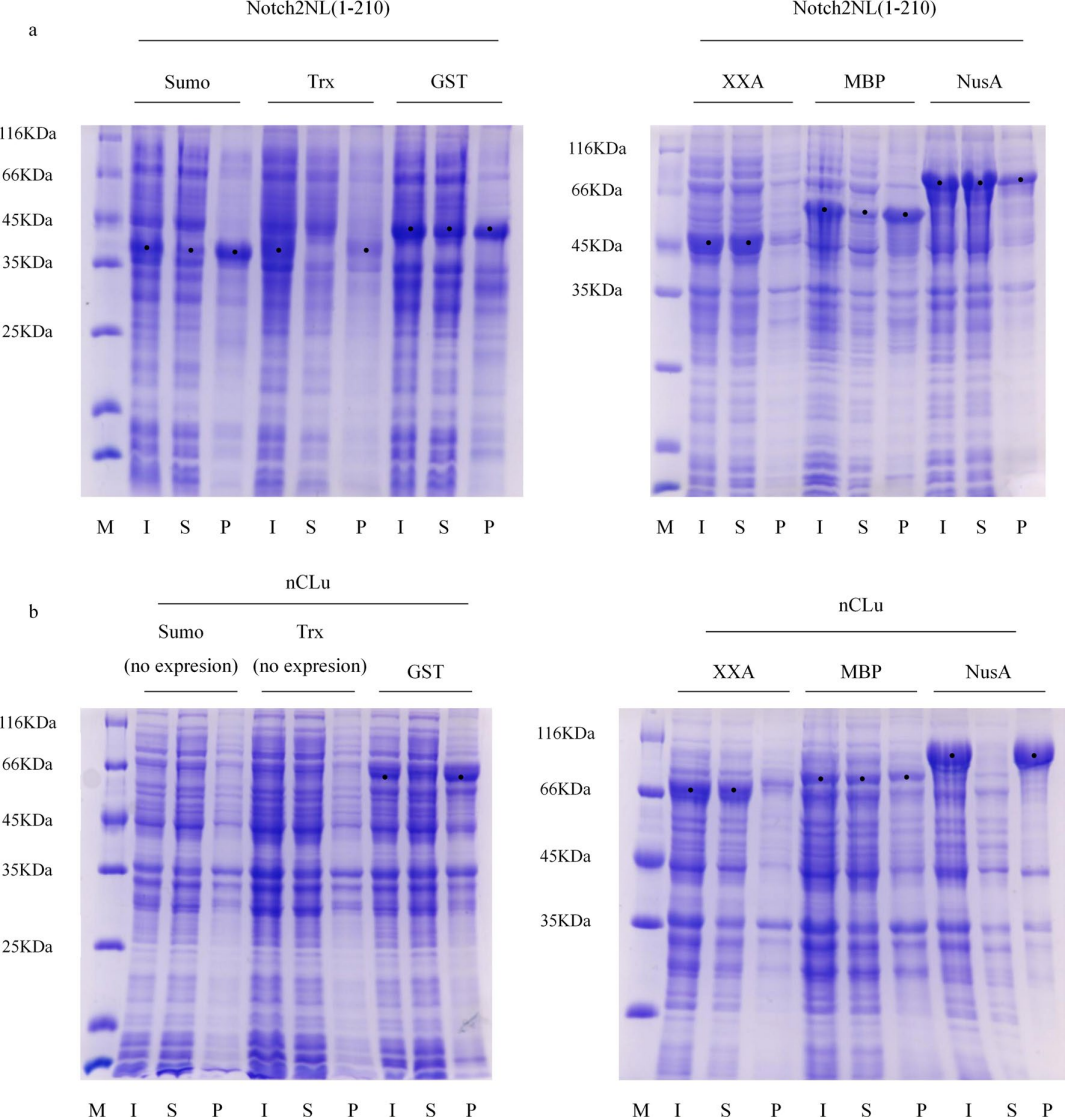
Multiple comparisons of the solubilizing effect of XXA and other tags on Notch2NL(1–210). We used six tags—sumo, Trx, GST, XXA, MBP, NusA—to conduct a lateral comparison of the solubilizing effect of Notch2NL (1–210). The results in the figure showed that sumo, Trx, and MBP had a poor solubilizing effect on Notch2NL(1–210), with most of the fusion protein existed in the precipitate, where the effect of GST was slightly better. The fusion effect of XXA and NusA were the best, with most of the fusion proteins expressed in soluble form. The solubility of each fusion protein was calculated by band intensity and is shown in Table 1. **b** We used six tags—sumo, Trx, GST, XXA, MBP, NusA—to conduct a lateral comparison of the solubilizing effect of nClu. The results in the figure showed that two fusion proteins, Sumo-nClu and Trx-nClu had not obvious expression. Meanwhile, GST and NusA had poor solubilization effect on nClu, with most of the fusion protein existed in the precipitate. XXA had best solubilization effect on nClu. The solubility of each fusion protein was calculated by band intensity and is shown in Table 1

In the previous experiments, a series of proteins that are difficult to make soluble when expressed were used to initially demonstrate that XXA is more effective than the commonly used solubilizing fusion tags. To further compare the solubilization effect of XXA and other tags more systematically, Notch2NL (1–210) and nClu were fused with Sumo, Trx, GST, XXA, MBP, and NusA separately, and we determined if they were soluble with each fusion protein (Fig. 5). Finally, the percentage of soluble expressed protein to total expressed protein was calculated by band intensity to show the solubilization effect of each tag more intuitively.

Our results showed that the two tags, Sumo and Trx had poor solubilization effect on Notch2NL (1–210), and the soluble recombinant protein accounted for less than 30% of the total recombinant protein. The effect of GST and MBP was better, with the soluble ratio less than 50%. NusA had more better solubilization effect, and the soluble ratio of fusion protein was 76%. XXA had best solubilization effect, and the soluble ratio of fusion protein was 94% (Fig. 5a and Table 1).

**Table 1:** The soluble level of Notch2NL(1–210) and nClu fused with different tags

Fusion tag (sequence length)	Solubility of fusion protein (%)	
	Notch2NL(1–210)	nClu
Sumo (98 aa)	24	No obvious expression
Trx (109 aa)	18	No obvious expression
GST (220 aa)	43	26
XXA(192 aa)	94	86
MBP(357 aa)	36	58
NusA(495 aa)	76	18

The results exhibited that two fusion proteins, Sumo-nClu and Trx-nClu had not obvious expression. Meanwhile, GST and NusA had poor solubilization effect on nClu, and the soluble recombinant protein accounted for less than 30% of the total recombinant protein. The effect of MBP was better, with the soluble ratio less than 60%. XXA had best solubilization effect, and the soluble ratio of fusion protein was 86% (Fig. 5b; Table 1).

Moreover, XXA has a shorter sequence length of 192 amino acids (aa) and the MBP sequence used was longer (357 aa) and the NusA sequence used was longest (495 aa). Thus, XXA had less effect on the yield of Notch2NL (1–210) and nClu.

### The tested inclusion bodies could be purified after being fused with XXA

**Fig. 6:**
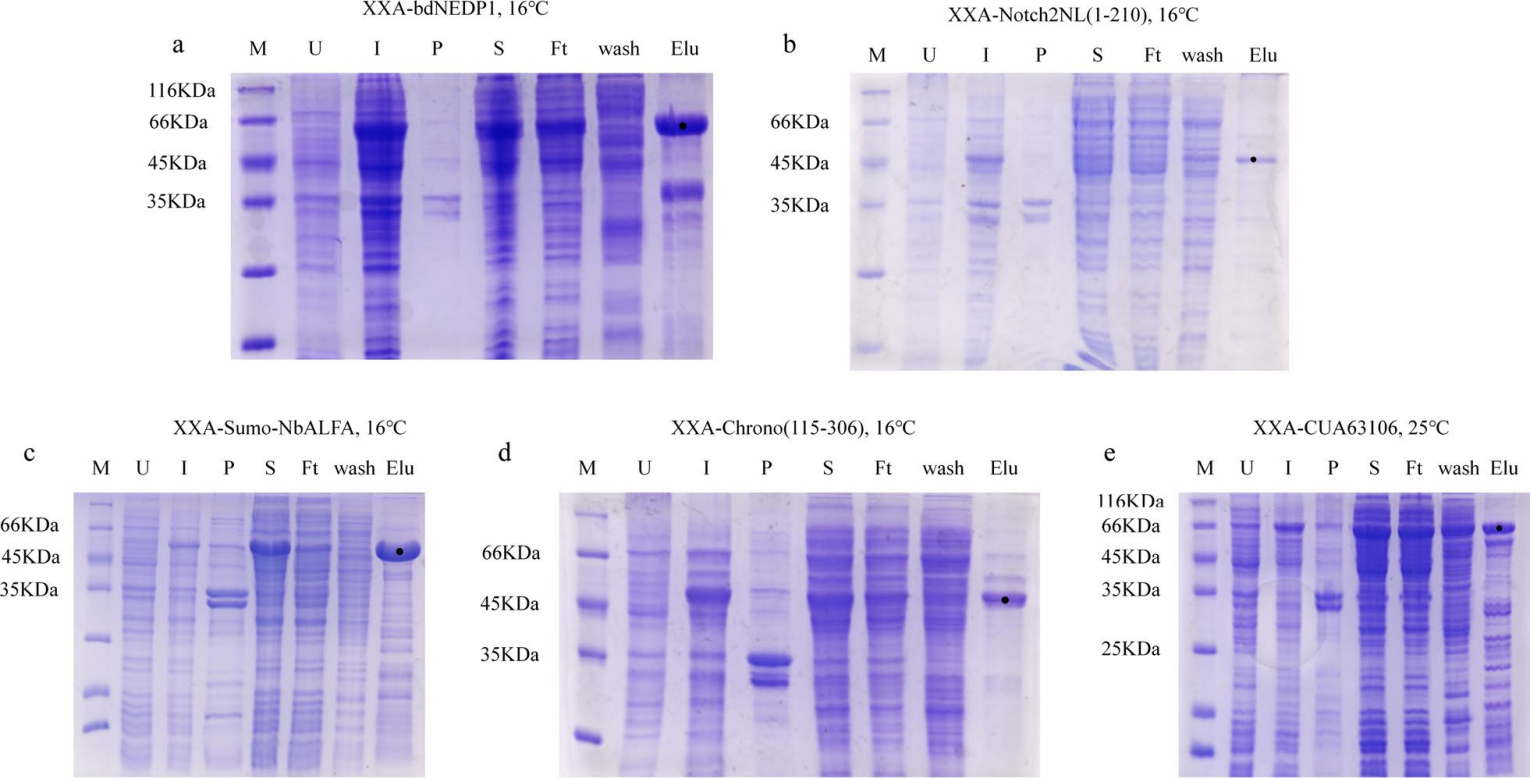
Several inclusion bodies that had enhanced soluble expression by XXA could bind the Ni^2+^ resin and be purified. **a** The purified result of fusion protein XXA-bdNEDP1. **b** The purified result of fusion protein XXA-Notch2NL(1–210). **c** The purified result of fusion protein XXA-sumo-NbALFA. **d** The purified result of fusion protein XXA-Chrono(115–306). **e** The purified result of fusion protein XXA-CUA63106. *M* marker, *U* uninduced whole cells, *I* induced whole cells, *S* supernatant of the induced cells, *P* precipitate of the induced cells, *Ft* flow through, *Wash* wash the impurity, *Elu* elution

Some heterologous proteins that contain poly His tag and are produced in *E. coli* could expressed in soluble form. However, purifying and obtaining of these recombinant proteins remains a difficult task due to their inability to be combined with metal ion affinity chromatography resin. Therefore, it was important to assess whether the inclusion body proteins used in the previous experiments could be purified by affinity chromatography after fusion with XXA. In this experiment, fusion proteins were tagged with poly His and XXA, and purified using Ni^2+^ resin. The results showed that the fusion proteins that were expressed at the low temperature of 16 °C and could be purified using Ni^2+^ resin to obtain pure samples, including XXA-bdNEDP1 (Fig. 6a), XXA-Notch2NL (1–210) (Fig. 6b), XXA-Sumo-NbALFA (Fig. 6c), and XXA-Chrono (115–306) (Fig. 6d), but not XXA-nClu. The fusion protein XXA-CUA63106 that was expressed at a high temperature could also be purified; however, the samples were not pure (Fig. 6e).

To avoid the interference of tags, proteins without any tags are required for many experiments. We tested whether the three commonly used specific proteases, TEV, sumo protease, and HRV_3C, could cleave recombinant proteins that were fused with XXA tags. To test this, TEV restriction sites were inserted into a XXA-bdNEDP1 fusion protein, the sumo that was derived from *Brachypodium distachyon* was inserted in XXA-Sumo-NbALFA fusion protein, and HRV_3C restriction sites were inserted into XXA-Chrono (115–306), XXA-Notch2NL (1–210), and XXA-CUA63106 when we constructed clones. Next, we used TEV, bdSENP1, and HRV_3C to digest the corresponding fusion proteins, and the samples were analyzed by SDS-PAGE. The results showed that all fusion proteins were successfully cleaved. This suggests that XXA does not affect the digestion of specific proteases to the fusion protein and is suitable as a solubilizing fusion tag. Meanwhile, the target protein remained in a soluble form and without re-precipitation after digestion (Additional file 3). Only the Notch2NL (1–210) band was greatly weakened after removing the tag, which may be caused by its own instability.

### The tested inclusion bodies fused with XXA may be functional

To confirm whether the inclusion body proteins used in the previous experiments were functional after being expressed in a soluble form, we purified the fusion proteins XXA-bdNEDP1 and XXA-NbALFA, and tested them using enzymatic digestion and western blot.

**Fig. 7:**
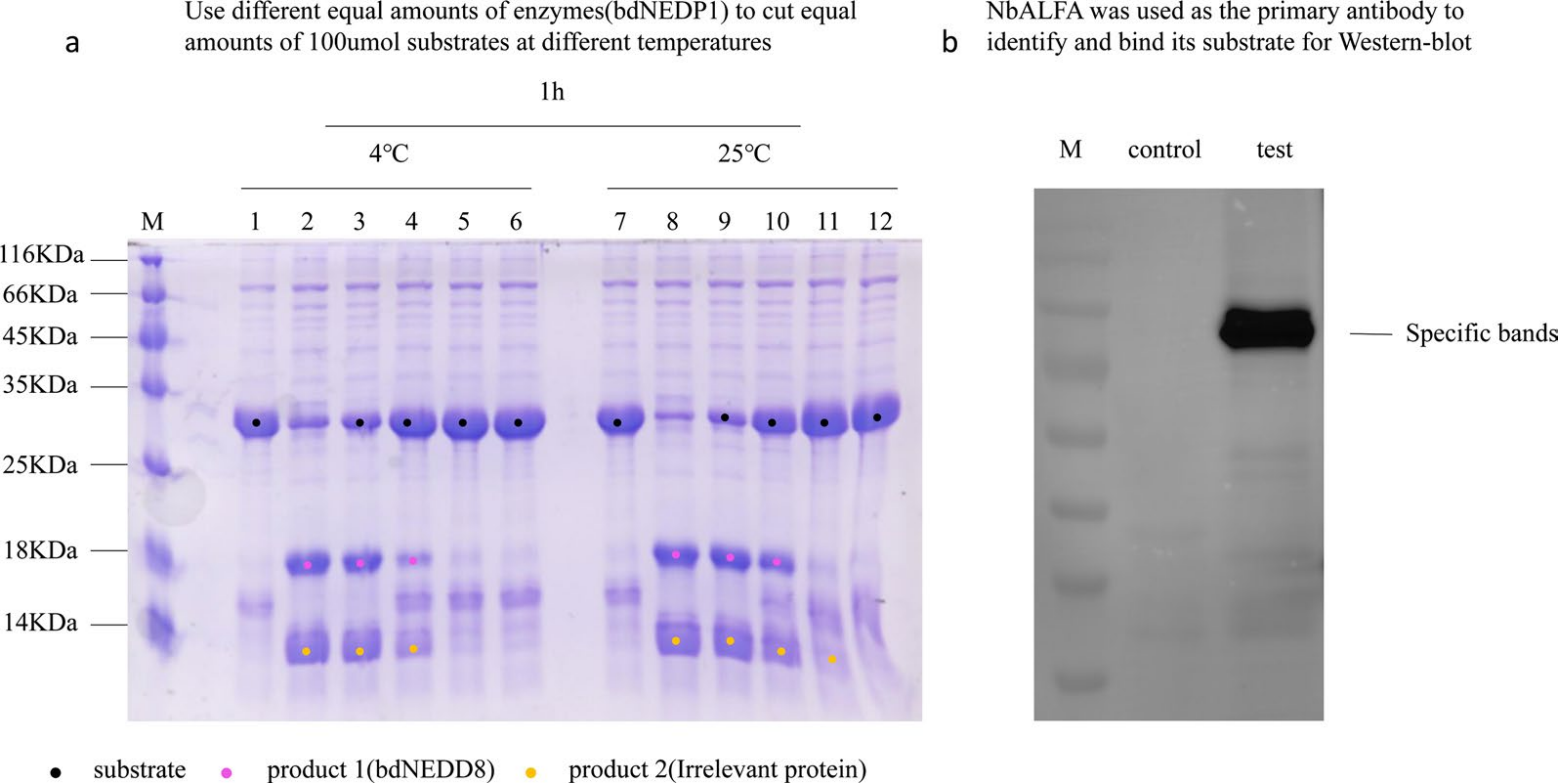
Functional verification of fusion proteins XXA-NbALFA and XXA-bdNEDP1. **a** The purified bdNEDP1 enzyme that fuses expression with the XXA tag was used to digest the substrate to test whether bdNEDP1 was active. The amount of substrate was fixed at 100 μmol, with lanes 1 and 7 being the control group without the enzyme. Ten μmol bdNEDP1 was used in the reactions of lanes 2 and 8, 1 μmol bdNEDP1 was used in the reactions of lanes 3 and 9, 100 nmol bdNEDP1 was used in the reactions of lanes 4 and 10, 10 nmol bdNEDP1 was used in the reactions of lanes 5 and 11, and 1 nmol bdNEDP1 was used in the reactions of lanes 6 and 12. The digestion reactions in lanes 1 to 6 was performed at 4 °C, while the reactions in lanes 7 to 12 were performed at 25 °C. The reaction time was fixed at 1 h. Whether bdNEDP1 was functional was judged by the reduction of the substrate and the increase in products. **b** The purified NbALFA nanobody that fuses expression with the XXA tag was used as the primary antibody to recognize its antigen to test whether NbALFA was functional. The substrate of the experimental group was *Aspergillus flavus* expressing the ALFA-tagged protein, while the control group was *Aspergillus flavus* not expressing the ALFA-tagged protein (empty vector control). The presence of a positive band that only exists in the experimental group indicates specificity, verifying that NbALFA was functional

As bdNEDP1 is a specific protease, its activity could be tested using enzymatic digestion. BdNEDP1 can recognize and cut the fusion protein that contains its specific substrate protein bdNEDD8 to produce two smaller proteins. The activity of bdNEDP1 was observed based on the decrease of the substrate and the increase of the product. The fusion protein that contained and used bdNEDD8 as a substrate was purified before use (Additional file 4). The results showed that bdNEDP1 had obvious enzymatic activity in the nmol level even at 4 °C (Fig. 7a).

NbALFA is an antibody that specifically recognizes the ALFA tag. Therefore, we used the fusion protein that contained the ALFA tag as the substrate and purified NbALFA as the primary antibody for western blotting. The activity of NbALFA was observed by whether the polyvinylidene fluoride (PVDF) membrane had the specific positive band. The western blot showed that NbALFA, solubilized by XXA, was functional because specific positive bands were observed, suggesting that the substrate, which was fused with ALFA, was successfully recognized (Fig. 7b).

## Discussion

Traditional solubilizing fusion tags show a trend that the solubilizing effect increases with increasing molecular weight. For example, the solubilizing effect of NusA and MBP is better than that of Trx and sumo. However, the yield of target protein will be affected by fusion tags with larger molecular weights, especially for small target proteins. However, the new solubilizing fusion tags developed in these experiments are based on AFPs and retro-proteins, with the aim of circumventing this issue. XXA is not only suitable for a series of insoluble proteins, but also has the best effect, compared with other tag proteins. Meanwhile, the sequence of XXA is only 192 aa, which has less of an effect on the yield of target proteins, but has a stronger solubilization effect than MBP and NusA. Thus, XXA is expected to truly achieve simultaneous enhance soluble expression and production.

Given its good performance, we are curious about how XXA achieves the mechanism of promoting soluble protein expression. A popular research area in the development of solubilizing fusion tags are molecular chaperones; hence, we were curious whether the artificial protein XXA acts as a molecular chaperone. Molecular chaperones that exert their function as co-expression factors are more common when recombinant proteins are expressed [28]. Therefore, we co-expressed XXA-pGex6p1 and nClu-pET50b to observe whether XXA could promote the soluble expression of nClu. The results showed that the co-expression of XXA could not facilitate the soluble expression of inclusion bodies, and the fusion protein NusA-nClu which was expressed by nClu-pET50b vector remain located in the precipitate (Additional file 5). This result is significantly different from the fusion expression in the previous experiment; therefore, XXA may not be a molecular chaperone.

XXA is a new artificial protein that does not exist in nature. Therefore, we proposed a reasonable conjecture on the mechanism of XXA based on the current work of this study and knowledge about proteins. Briefly, we think the effect of XXA as a solubilizing fusion tag may be related to the formation of oligomers and the distribution of surface charge.

**Fig. 8:**
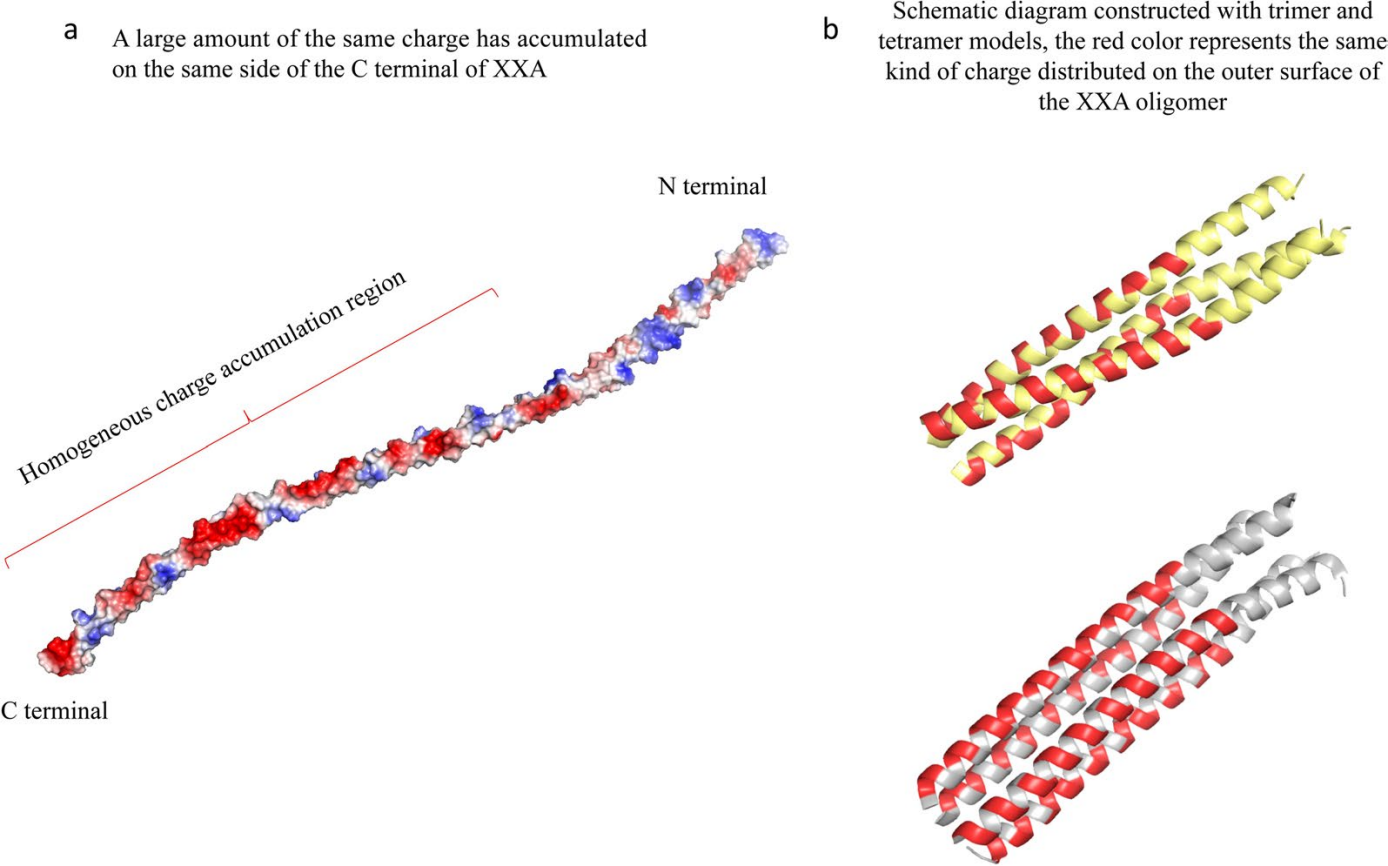
Charge distribution and oligomer model of XXA.** a** The charge distribution of the XXA monomer was analyzed using the tertiary structure from tFold. **b** A schematic diagram was constructed to show the trimer and tetramer models of XXA. Red color represents that the same kind of charge was distributed on the outer surface of the XXA oligomer

In previous experiments, we found that the secondary structure of XXA is mainly composed of α-helices and exists as oligomers in aqueous solution. Coiled coil domain is a common way to form oligomers in proteins, and we found that the C-terminus of XXA is predicted to form coiled coils (Additional file 6). The hydrophobic core of a coiled coil is embedded in the interior to mediate the binding between monomers [29]. We noticed that a large amount of the same charge was accumulated at one side of the C-terminus in the XXA monomer, while the other side was a hydrophobic region (Fig. 8a). A schematic diagram of XXA oligomers shows that hydrophobic regions are embedded and form the core of the polymer while the side chains of the same charge region are distributed on the surface (Fig. 8b).

**Fig. 9:**
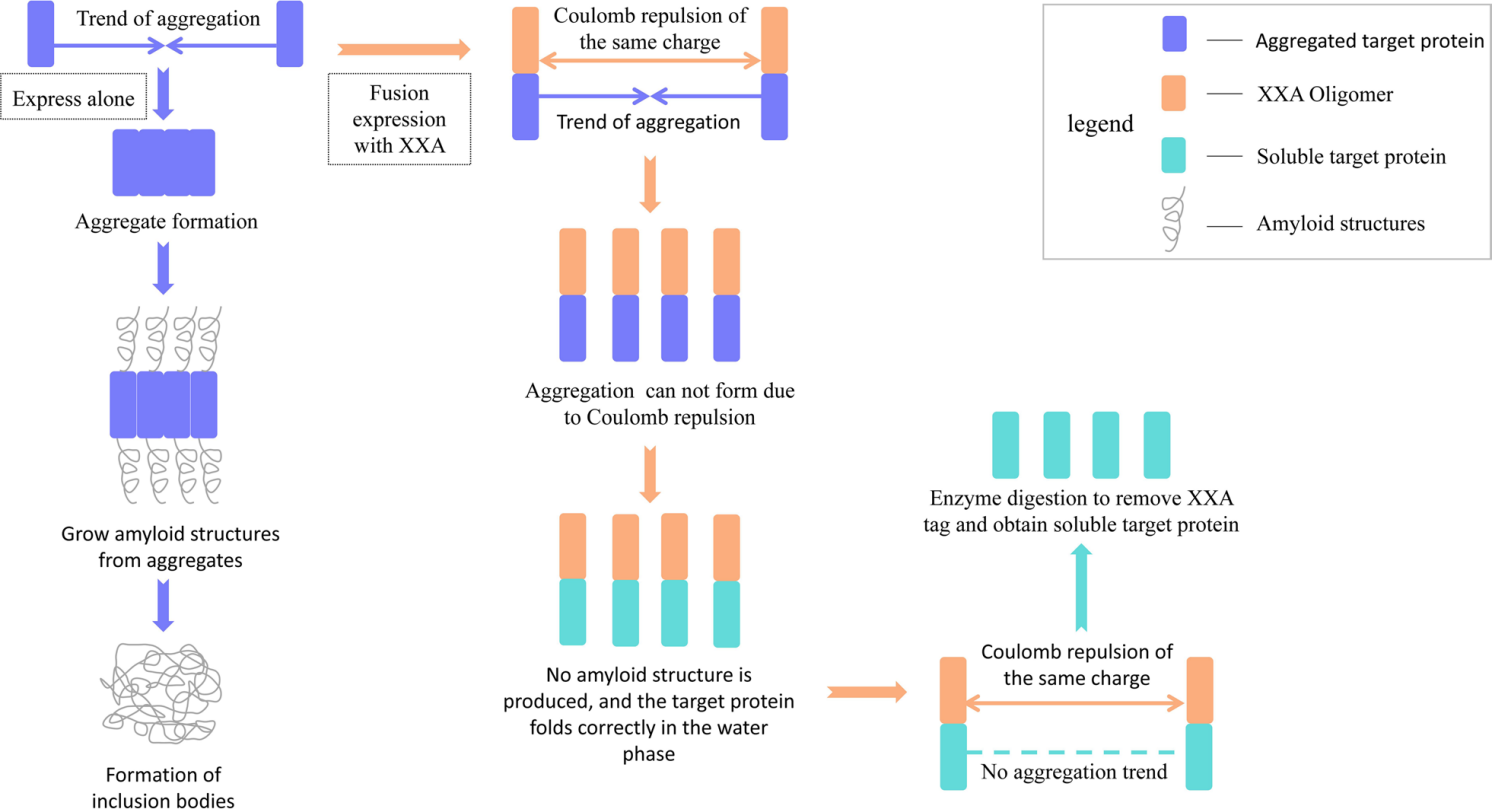
Conjecture diagram of the principle solubilizing effect of XXA. The aggregation tendency of the target inclusion body protein is prevented by strong Coulomb repulsion of the XXA oligomer. Therefore, the fusion protein cannot aggregate. As a result, amyloid structures cannot form because the aggregation of the target protein is prevented. The target protein can fold correctly in the water phase, significantly improving the soluble expression of the inclusion bodies

The inclusion bodies are closely related to the occurrence of amyloid structures, and the amyloid structures tend to grow initially from protein aggregation [30]. The amyloid structures are infectious and will further produce a large amount of precipitation to form inclusion bodies. We think the large of amount of the same charge, located on the surface of the XXA oligomer, is the key factor to enhance protein soluble expression. The aggregation tendency of the target inclusion body protein is prevented by strong Coulomb repulsion of the XXA oligomer. Therefore, the fusion proteins cannot aggregate with each other. As a result, amyloid structures cannot form, because the aggregation of the target protein is prevented, and the target protein can fold correctly in the water phase, which significantly improves the soluble expression of the inclusion bodies (Fig. 9).

We speculate that some optimizations could be performed with the XXA tag following three points based on our experience. The first is that a GST tag can be co-fused with the XXA tag, in order to purify the fusion protein using the GST affinity resin. The second is that the XXA tag can be fused both in the N- and C-terminal of the target protein to enhance the solubilizing effect. The third is to further modify its sequence based on the knowledge of crystallography and biochemistry.

In summary, we developed a new remarkable solubilizing fusion tag to enhance the soluble expression of inclusion bodies. This is the first time that AFPs have been introduced into the development of solubilizing fusion tags, and it is also the first time that retro-proteins have been put into practical use.

## Conclusion

Artificial protein XXA is a retro-protein (reverse sequence) derived from AFP AXX. Their full length sequences are 192 aa and both XXA and AXX showed excellent ability to enhance the soluble expression for inclusion bodies as a solubilizing fusion tag. However, XXA showed more stability and better expression than AXX.

Through a series of experiments, we not only have verified that XXA could clearly enhance the soluble expression of many inclusion bodies, but also showed its solubilizing capacity is better than a series of commonly used tags. We also verified that these soluble recombined proteins obtained by fusion with the XXA tag can be purified, digested, remain functional, and even remain soluble after removal of the XXA tag. Finally, a hypothesis of the mechanism behind XXA’s promotion of protein soluble expression was proposed and possible improvements were suggested based on the results of these experiments and the existing knowledge of protein biochemistry.

## Materials and methods

### Amino acid sequence

AXX:

MQDESLADKAKSAIETAKHAVSDAAQKVKETVTGAAADVQETARDVTQDQRQNLGYAEQKAADTLGDVKAAAQEAYESAKQRASEAAEGAKSTASELGGSAERAVRDAAGGAEGAGRDAQGAAREGLKGAEGAGATDEARRHAEDVADTAKEKYSELKGDAKEGLGRAQAKGEDLAGDASKAAQDAADRLKP.

XXA:

PKLRDAADQAAKSADGALDEGKAQARGLGEKADGKLESYKEKATDAVDEAHRRAEDTAGAGEAGKLGERAAGQADRGAGEAGGAADRVAREASGGLESATSKAGEAAESARQKASEYAEQAAAKVDGLTDAAKQEAYGLNQRQDQTVDRATEQVDAAAGTVTEKVKQAADSVAHKATEIASKAKDALSEDQM.

### Protein and gene analysis

Sequence information of all proteins and genes were collected from NCBI. The UniProt database was used in addition to NCBI, when BLASTing the protein and nucleic acid sequences. The protein molecular weight, theoretical pI, aliphatic index, grand average of hydropathicity, and other information were calculated using the ProtParam tool (https://web.expasy.org/protparam/). The protein secondary structure information was predicted using the NPS@_GORIV server (https://npsa-prabi.ibcp.fr/cgi-bin/npsa_automat.pl?page=npsa_gor4.html). The tertiary structure was predicted using the tfold tool [31](https://drug.ai.tencent.com/console/cn/tfold?type=predict) and all structural figures were prepared by PyMOL. The signal peptide information was predicted using SignalP 5.0 (https://services.healthtech.dtu.dk/service.php?SignalP-5.0) and the transmembrane region information was calculated using the TMHMM server v.2.0 (https://services.healthtech.dtu.dk/service.php?TMHMM-2.0). The coiled coil information was predicted using the Coils server (https://embnet.vital-it.ch/software/COILS_form.html).

### Strains and clone

All cloning work was performed using the XL1blue strain, and protein expression was performed with the BL21 (DE3) strain. Gene amplification followed standard PCR procedures and clone construction used standard double enzyme digestion. But clones of multiple tags comparison experiments were constructed by ligase independent cloning (LIC) method [32] and the gene of XXA was replaced with other tag’s genes. All clone-related enzymes and buffers were purchased from TaKaRa (Dalian, China). The pET series of vectors were obtained from Novagen (Germany), the pCold series of vectors were purchased from TaKaRa, and the pGEX series of vectors were obtained from GE Healthcare (USA).

The pXXA vector was modified from the pET series of vectors. A gene that contains 8 × His, Flag, XXA tag, and PreScission site was inserted between BamH1 and Ecor1. Meanwhile, the target protein gene with stop codon was inserted between Ecor1 and Xho1, but the insertion of the nClu gene used the Hind3 site to replace Ecor1.

### Construction of the pXXA vector

To make it more convenient to compare the effect of XXA with other common solubilizing fusion tags, we constructed a pXXA vector based on the T7 promoter system that contained the XXA gene. After the start codon of the pXXA vector, there is a 8 × His tag, a Flag tag, an XXA tag, PreScission (HRV_3C) site, target protein insertion site, and stop codon arranged in sequence from the 5′ to 3′ end. (Additional file 7). The 8 × His tag enable easier purification of recombinant proteins, the Flag tag is used to detect the expression by western blot, the XXA tag was used to improve soluble expression for the target protein, and the PreScission site was used to remove other elements besides the target protein. Finally, the expression process is terminated in time at the stop codon behind the target protein.

### Expression and purification

The pGex6p1 vector was used for both AXX and XXA. The fusion expression of the XXA tag with Chrono (115–306), Notch2NL (1–210), nClu, NbALFA, and CUA63106 used the pXXA vector. The target protein gene was inserted into the restriction site downstream of the XXA gene during cloning. The pColdI vector was used for bdNEDP1 and fusion proteins with XXA. The XXA tag was located upstream or downstream of the bdNEDP1 gene; meanwhile, a TEV site existed between XXA and bdNEDP1. The pGex6p1, pET32a, pET50b vector was used to express the fusion protein GST-Chrono (115–306), Trx-Notch2NL (1–210), or NusA-nClu. The expression of fusion protein XXA-Sumo-NbALFA used the pET28 vector. The XXA gene was inserted between BamH1 and Ecor1, the Sumo gene was inserted between Sal1 and Hind3, and NbALFA was inserted between Hind3 and Xho1.

The proteins were expressed by BL21(DE3) at a low temperature of 16 °C without unique instructions. *E. coli* was induced with 0.5 mM IPTG for 24 h when the OD_600_ was 0.6. The cell lysate was purified after lysis (50 mM Tris–HCl, 300 mM KCl, 5% glycerol, pH 8.0, 1 mM PMSF), sonicated, and centrifuged, before being incubated with Ni^2+^ affinity beads (GE Healthcare) and washed using lysis buffer containing 60 mM imidazole. Lastly, the protein was eluted with lysis buffer containing 500 mM imidazole. The expression of fusion protein MBP-CUA63106 and XXA-CUA63106 was induced with 0.5 mM IPTG at 25 °C for 12 h, and the purification was performed with the same buffer and procedure using Ni^2+^ resin.

When XXA and AXX were expressed alone, they were expressed at 16 °C and purified used GST resin. *E. coli* were induced by 0.5 mM IPTG for 24 h when the OD_600_ was 0.6. The cell lysate was purified after lysis (50 mM Tris–HCl, 300 mM KCl, 5% glycerol, pH 8.0, 1 mM PMSF), sonicated, and centrifuged, before being incubated with GST-affinity beads (GE Healthcare) and washed with lysis buffer. Last, the protein was eluted by lysis buffer containing 10 mM reduced glutathione.

The method for calculation of the protein solubility was according to the literature [4].

### Removal of tags

The Sumo protease was used to remove the XXA-Sumo tag of fusion protein XXA-Sumo-NbALFA. The TEV enzyme was used to remove the XXA tag of fusion protein XXA with bdNEDP1. The PreScission protease was used to remove the XXA tag of other fusion proteins. The digestion reaction for removing the tag was performed at 4 °C for 12 h, and the digestion reaction was simultaneously dialyzed into lysis buffer containing no imidazole or no reduced glutathione. After the digestion was completed, Ni^2+^ resin or GST resin was used to remove the tag and obtain the target protein without the tag.

### Protein thermal shift assays

Thermal shift assays were conducted according to a previous study [33] and Thermo Fisher’s manual with minor modifications. XXA and AXX proteins at 1 mg/ml in 50 mM Tris–HCl, 300 mM KCl, pH 8.0 were incubated in 20 μl with 5 × SYPRO Orange (Sigma, Germany). Thermofisher QuantStudio 5 was used to conduct the experiment and fluorescence was detected over a temperature range of 15–95 °C. Excel was used to draw images based on the data and the experiment was repeated three times.

### Determination of bdNEDP1 and NbALFA activity

The constructed XXA-bdNEDP1-pCold1 vector and the BL21 (DE3) strain were used for expression. The cells were incubated in ice-water mixture for 30 min when the OD_600_ was 0.6. Next, IPTG was added to a final concentration of 0.5 mM for induction. The fusion protein was purified according to previous introduction of the method (see “Expression and purification”) after expression at 16 °C for 24 h. Next, the TEV enzyme was added for digestion at 4 °C for 12 h and dialyzed into lysis buffer simultaneously. The mixture was incubated with Ni^2+^ affinity beads after digestion and the XXA tag bound to Ni^2+^ affinity beads; thus the target protein bdNEDP1 flows through and exists in the lysis buffer. The substrate gene was fused by the bdNEDD8 gene with irrelevant protein gene (BC2-nanobody was used here), and bdNEDD8 was located in 5′ terminal. The substrate gene was cloned into the pColdI vector for expression at 16 °C and purified using Ni^2+^ resin for later use (Additional file 2). Functional BdNEDP1 will recognize bdNEDD8 and cut between bdNEDD8 and irrelevant protein to produce two smaller protein fragments. The digestion reaction was performed at 4 °C and 25 °C, using 10 μM, 1 μM, 100 nM, 10 nM, and 1 nM bdNEDP1 to digest the 100 μM substrate separately, and the digestion reaction time was fixed at 1 h. SDS-PAGE was used to detect the result of the reaction when the digestion reaction was completed.

The constructed XXA-Sumo-NbALFA-pet28 vector was transformed into the BL21 (DE3) strain. After expression at 37 °C for 3 h, the crude lysate was purified using Ni^2+^ resin. The purification conditions were as outlined above, and the NbALFA protein was obtained as the primary antibody. Wild type and the ALFA-chimeric versicolorin B synthase (ALFA-Vbs)-expressing *Aspergillus flavus* strain were used as substrate, which were kindly provided by Dr. Xin-Yi Nie (School of Life Sciences, Fujian Agriculture and Forestry University, Fuzhou, P.R. China). The crude lysates from these two strains were subjected to SDS-PAGE followed by western blot. The specific protein band, bound to the NbALFA, was detected using an anti-camelid VHH-rabbit IgG-HRP-linked antibody (Genescript, Nanjing). BeyoECL Star (Beyotime Biotechnology, Shanghai) was used for chemiluminescence and detection.

### Accession numbers

AXX: PRW45461.

Chrono: Q8N365.

Notch2NL: Q7Z3S9.

Clu: P10909.

bdNEDP1: XP_024311124.

bdNEDD8: XP_003578133.

BC2-nanobody: 5IVN_A.

CUA63106: CUA63106.

## Supplementary Information

**Additional file 1**: Physical and chemical properties of AXX and XXA.

**Additional file 2**: The predicted secondary structure of AXX and XXA. The secondary structures of XXA and AXX show they are mainly composed of long α-helices.

**Additional file 3**: The target protein remain soluble after removal of the XXA tag. **a** The TEV enzyme successfully digested the purified XXA-bdNEDP1 fusion protein. After the tag was removed, the bdNEDP1 remained located in the supernatant. **b** Sumo protease successfully cleaved the XXA-Sumo-NbALFA fusion protein and NbALFA remain located in the water phase after digest. **c** The fusion proteins XXACUA63106, XXA-Notch2NL (1–210), and XXA-Chrono (115–306) were successfully cleaved by PreScission to remove the solubilizing fusion tag. These three proteins remained soluble after removal of the XXA tag. M: Marker, U: Undigested, D: Digested, E: Enzyme, T: Target protein, X: XXA tag.

**Additional file 4**: The SDS-PAGE result of purified bdNEDD8-BC2 fusion protein. The bdNEDD8-BC2 fusion protein was purified using Ni affinity resin and used as substrate for the digestion reaction of bdNEDP1. M: Marker, U: Uninduced, I: Induced, S: Supernatant, P: Precipitation, Ft: Flow through, Wash: wash the impurity, Elu: Elution.

**Additional file 5**: Co-expression of XXA and nClu. XXA was distributed in the supernatant but the NusA-nClu fusion protein remain expression as inclusion body. Thus, the co-expressed XXA cannot promote the soluble expression of protein and it is likely not a molecular chaperone. M: Marker, U: Uninduced, I: Induced, S: Supernatant, P: Precipitation.

**Additional file 6**: The predicted Coiled Coil region of XXA. The predicted results showed that the C-terminal region of XXA could form the coiled coil domain.

**Additional file 7**: The composition diagram of pXXA vector. The pXXA vector that contains the XXA tag was modified from the pET series vectors and can be conveniently used to enhance soluble expression of heterologous proteins.

## Data Availability

All data generated or analyzed during this study are included in this published article (as well as its Additional files).

## Abbreviations

AFP: Antifreeze protein
IPTG: Isopropyl-β-d-thiogalactopyranoside
Tm: Melt temperature
PVDF: Polyvinylidene fluoride
BSA: Bovine albumin
His: Histidine
LIC: Ligase independent cloning
Aa: Amino acids
