# Supplementary figures and images for: Retro-protein XXA is a remarkable solubilizing fusion tag for inclusion bodies
Source: Microb Cell Fact. 2022 Apr 2;21:51. doi: 10.1186/s12934-022-01776-7 (PMC8977028; doi:10.1186/s12934-022-01776-7)

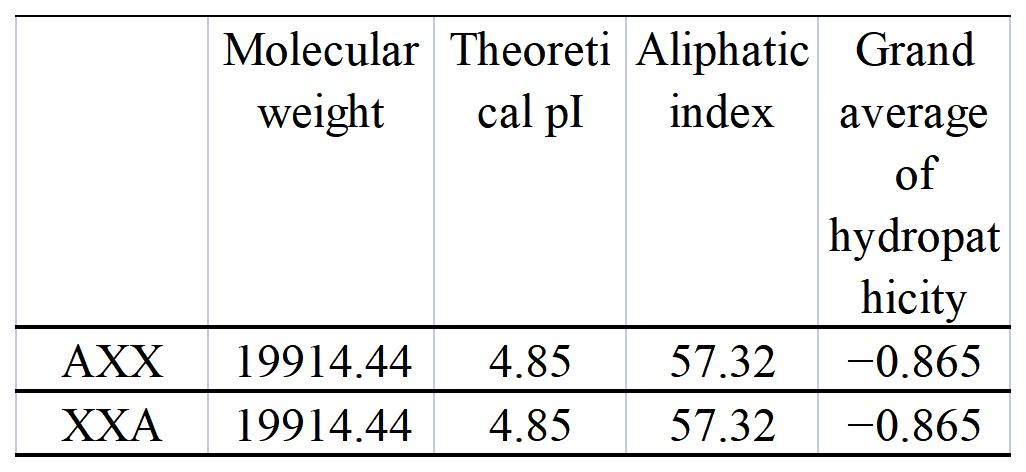

Supplement: Supplementary file 1 — Additional file 1: Physical and chemical properties of AXX and XXA. [file 12934_2022_1776_MOESM1_ESM.jpg]

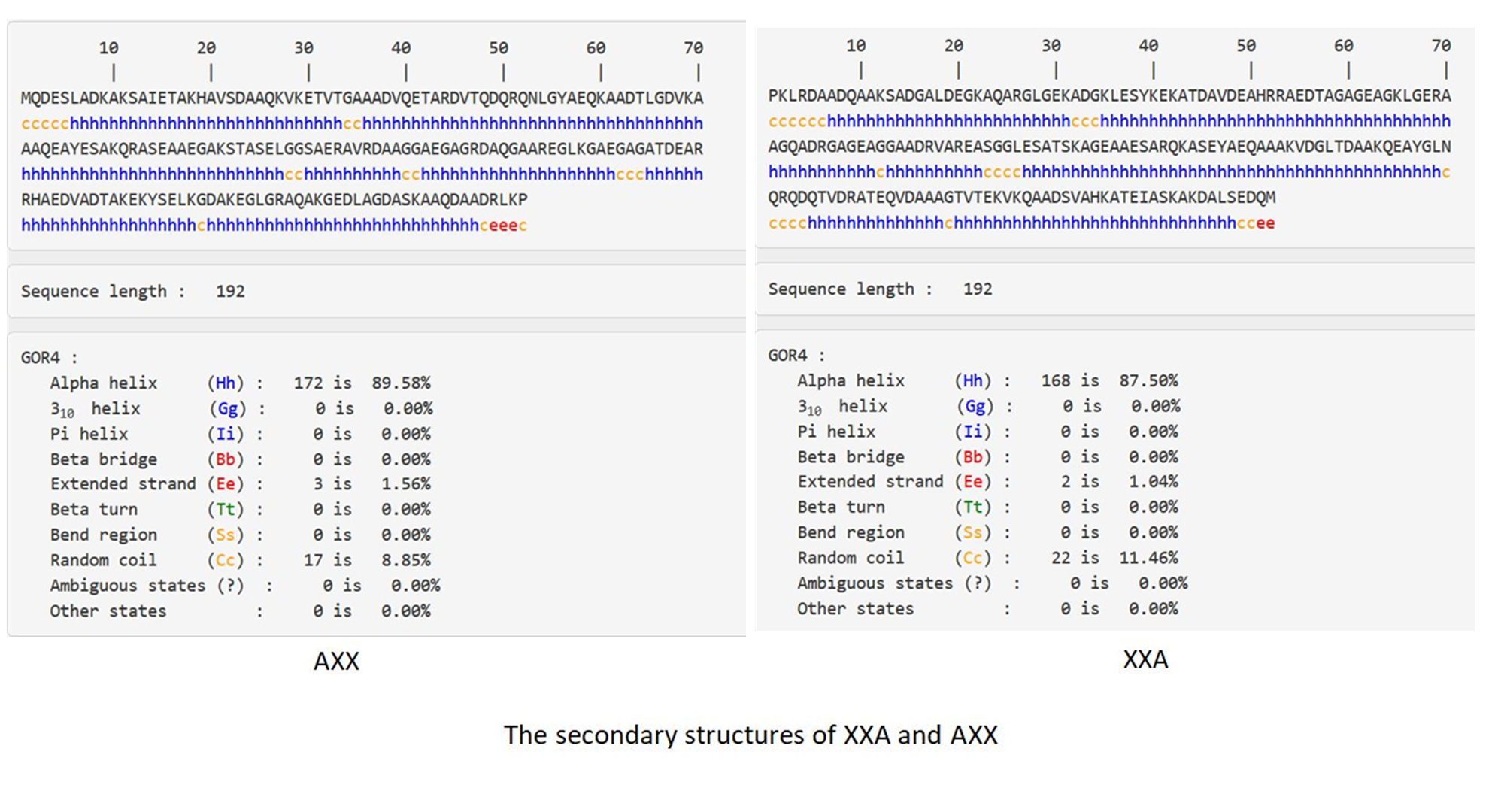

Supplement: Supplementary file 2 — Additional file 2: The predicted secondary structure of AXX and XXA. The secondary structures of XXA and AXX show they are mainly composed of long α-helices. [file 12934_2022_1776_MOESM2_ESM.jpg]

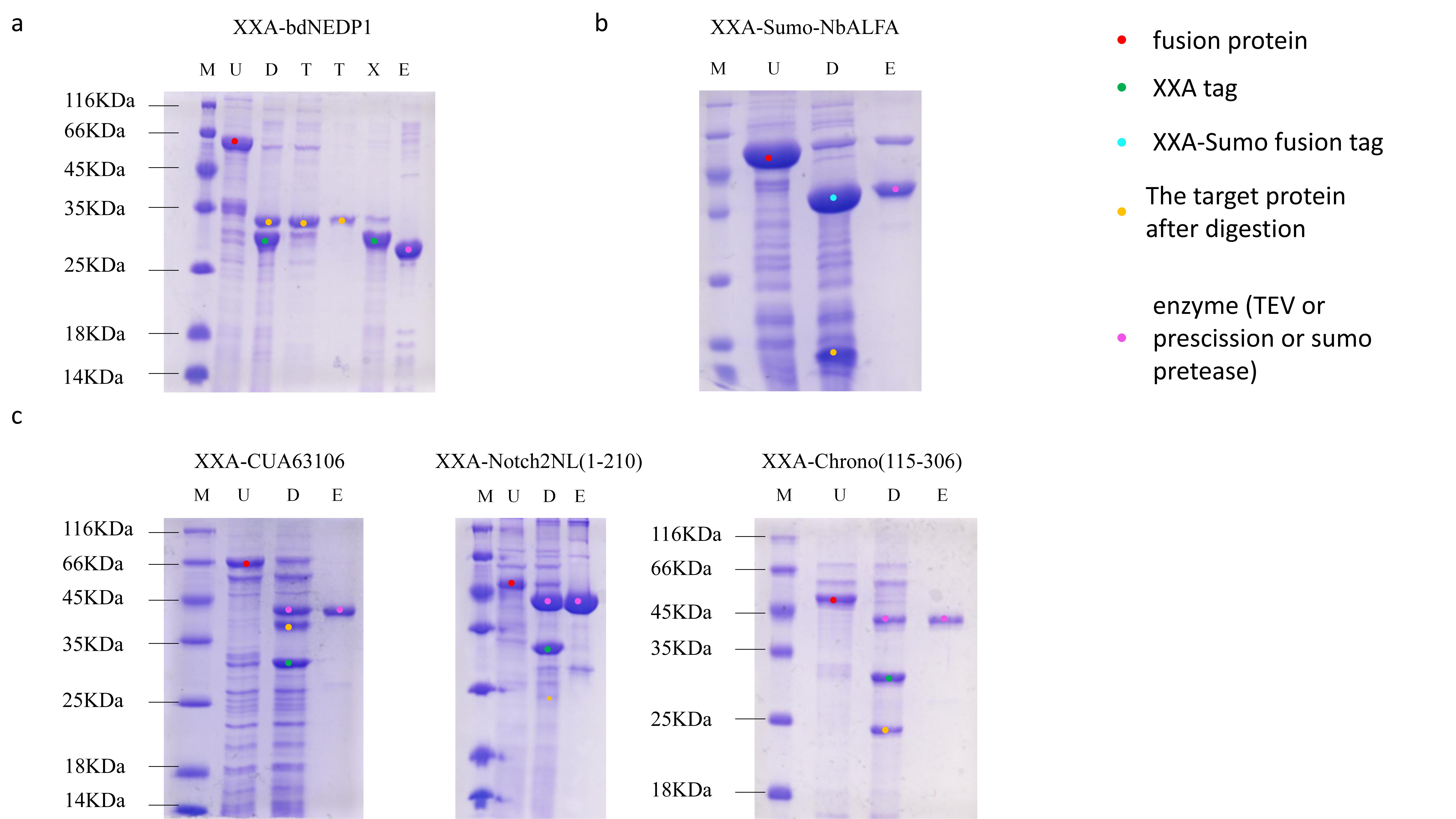

Supplement: Supplementary file 3 — Additional file 3: The target protein remain soluble after removal of the XXA tag. a The TEV enzyme successfully digested the purified XXA-bdNEDP1 fusion protein. After the tag was removed, the bdNEDP1 remained located in the supernatant. b Sumo protease successfully cleaved the XXA-Sumo-NbALFA fusion protein and NbALFA remain located in the water phase after digest. c The fusion proteins XXACUA63106, XXA-Notch2NL (1–210), and XXA-Chrono (115–306) were successfully cleaved by PreScission to remove the solubilizing fusion tag. These three proteins remained soluble after removal of the XXA tag. M: Marker, U: Undigested, D: Digested, E: Enzyme, T: Target protein, X: XXA tag. [file 12934_2022_1776_MOESM3_ESM.jpg]

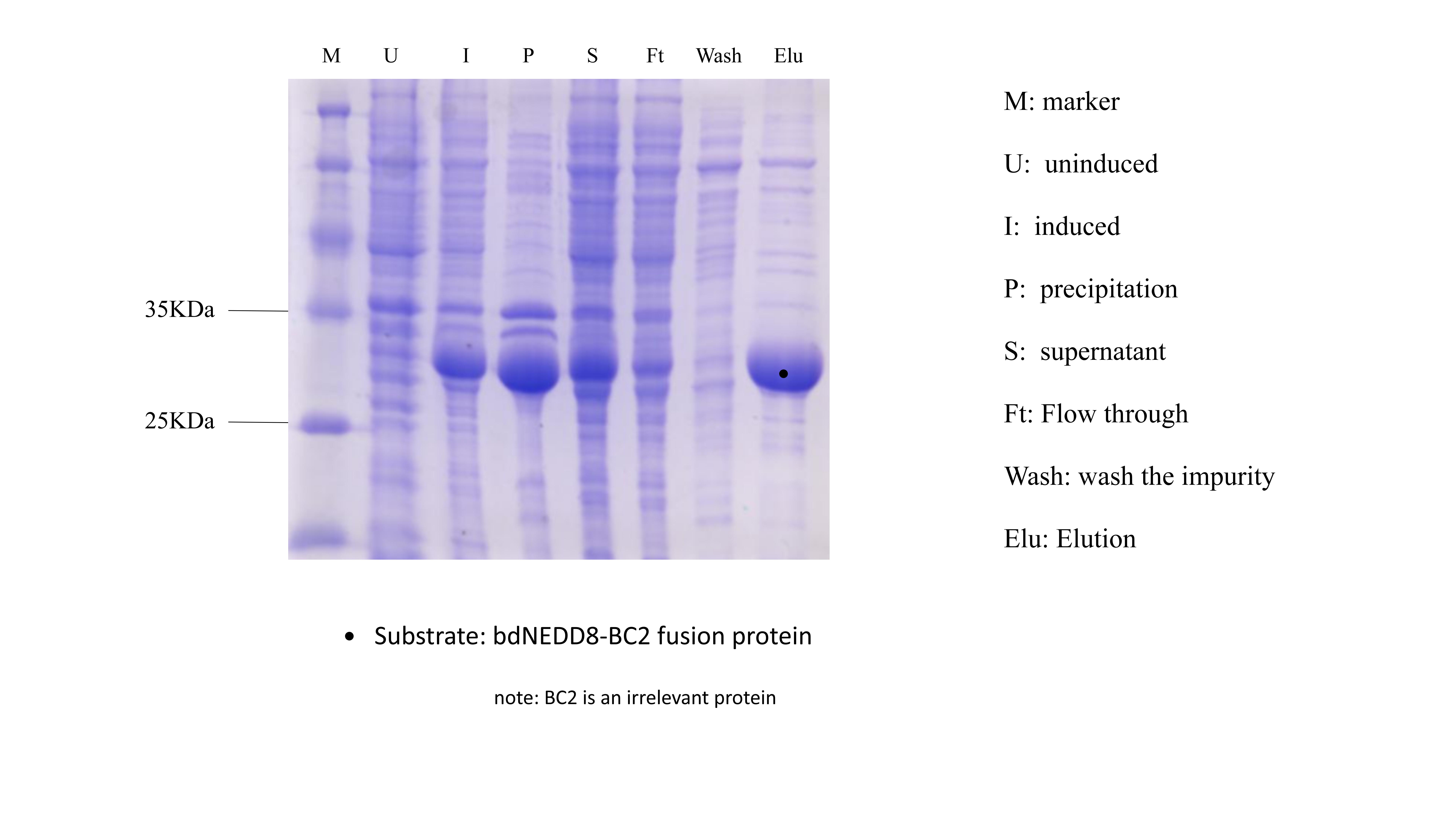

Supplement: Supplementary file 4 — Additional file 4: The SDS-PAGE result of purified bdNEDD8-BC2 fusion protein. The bdNEDD8-BC2 fusion protein was purified using Ni affinity resin and used as substrate for the digestion reaction of bdNEDP1. M: Marker, U: Uninduced, I: Induced, S: Supernatant, P: Precipitation, Ft: Flow through, Wash: wash the impurity, Elu: Elution. [file 12934_2022_1776_MOESM4_ESM.jpg]

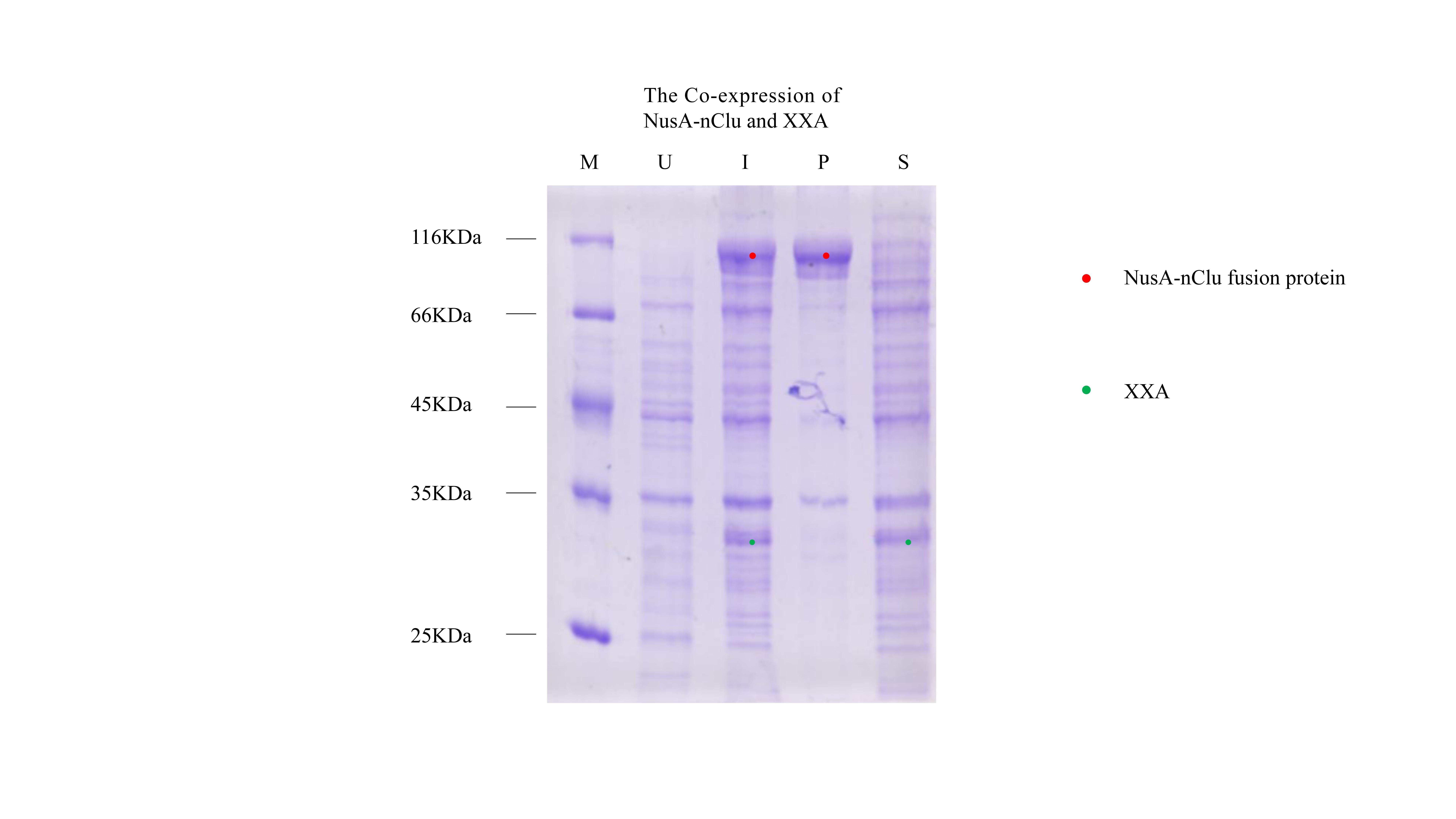

Supplement: Supplementary file 5 — Additional file 5: Co-expression of XXA and nClu. XXA was distributed in the supernatant but the NusA-nClu fusion protein remain expression as inclusion body. Thus, the co-expressed XXA cannot promote the soluble expression of protein and it is likely not a molecular chaperone. M: Marker, U: Uninduced, I: Induced, S: Supernatant, P: Precipitation. [file 12934_2022_1776_MOESM5_ESM.jpg]

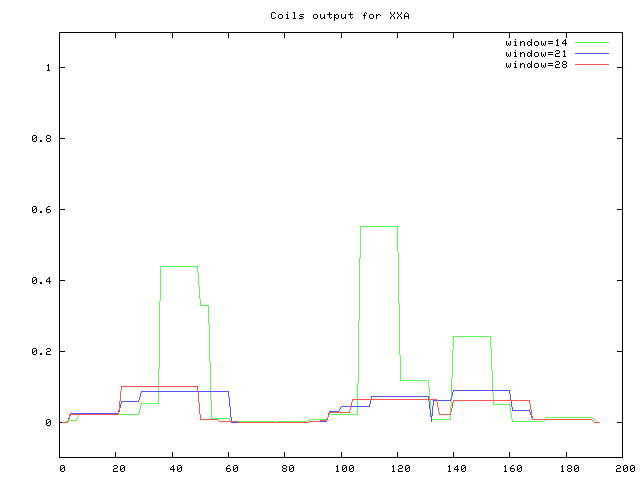

Supplement: Supplementary file 6 — Additional file 6: The predicted Coiled Coil region of XXA. The predicted results showed that the C-terminal region of XXA could form the coiled coil domain. [file 12934_2022_1776_MOESM6_ESM.png]

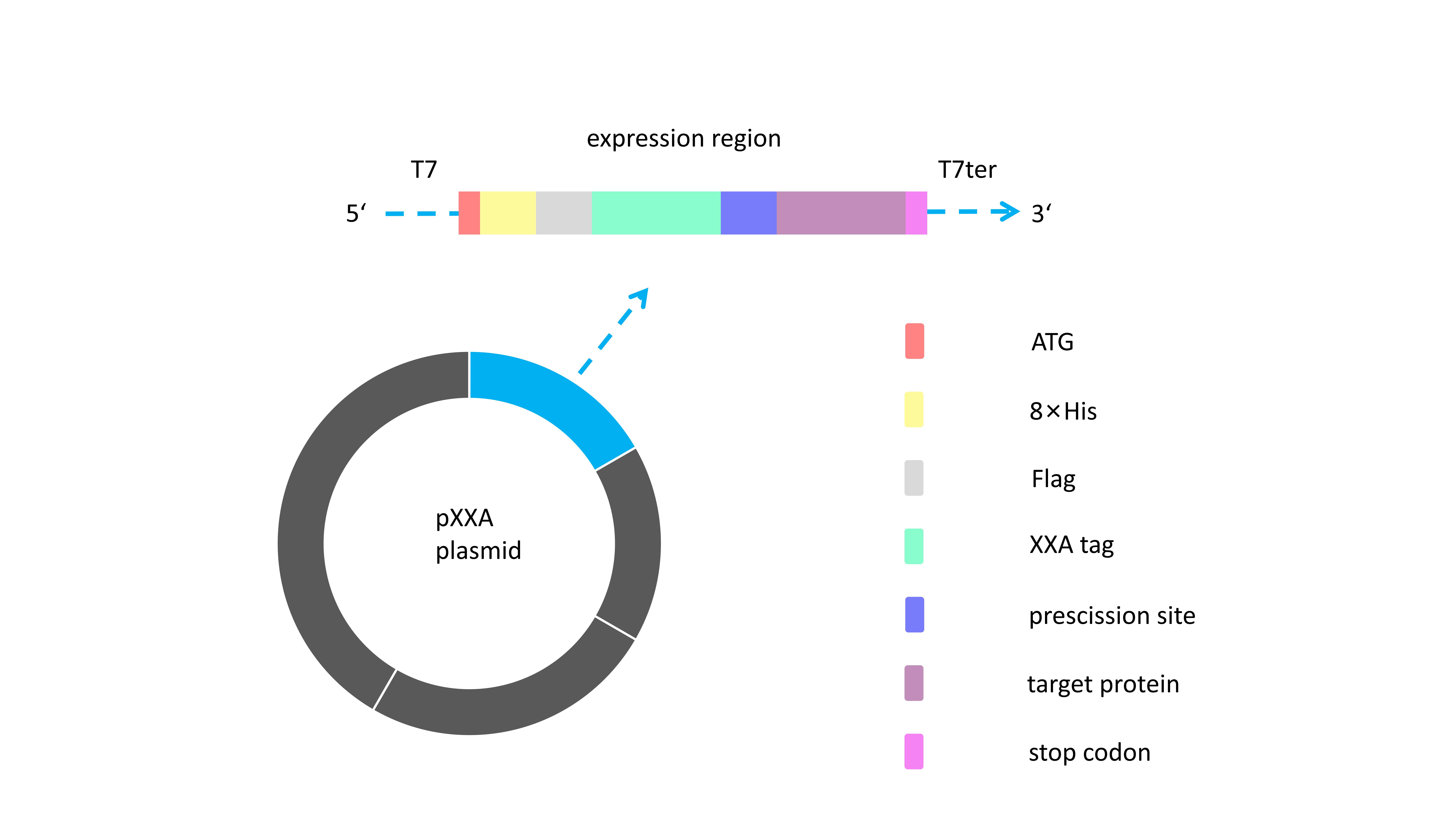

Supplement: Supplementary file 7 — Additional file 7: The composition diagram of pXXA vector. The pXXA vector that contains the XXA tag was modified from the pET series vectors and can be conveniently used to enhance soluble expression of heterologous proteins. [file 12934_2022_1776_MOESM7_ESM.jpg]
